# Is primary care a neglected piece of the jigsaw in ensuring optimal stroke care? Results of a national study

**DOI:** 10.1186/1471-2296-10-27

**Published:** 2009-04-29

**Authors:** David L Whitford, Anne Hickey, Frances Horgan, Bernadette O'Sullivan, Hannah McGee, Desmond O'Neill

**Affiliations:** 1 Department of Family & Community Medicine, Royal College of Surgeons in Ireland-Medical University of Bahrain, PO Box 15503, Adliya, Kingdom of Bahrain; 2 Department of Psychology, Royal College of Surgeons in Ireland, Dublin 2, Ireland; 3 School of Physiotherapy, Royal College of Surgeons in Ireland, Dublin 2, Ireland; 4 Department of Medical Gerontology, Trinity College Dublin, Dublin, Ireland

## Abstract

**Background:**

Stroke is a major cause of mortality and morbidity with potential for improved care and prevention through general practice. A national survey was undertaken to determine current resources and needs for optimal stroke prevention and care.

**Methods:**

Postal survey of random sample of general practitioners undertaken (N = 204; 46% response). Topics included practice organisation, primary prevention, acute management, secondary prevention, long-term care and rehabilitation.

**Results:**

Service organisation for both primary and secondary prevention was poor. Home management of acute stroke patients was used at some stage by 50% of responders, accounting for 7.3% of all stroke patients. Being in a structured cardiovascular management scheme, a training practice, a larger practice, or a practice employing a practice nurse were associated with structures and processes likely to support stroke prevention and care.

**Conclusion:**

General practices were not fulfilling their potential to provide stroke prevention and long-term management. Systems of structured stroke management in general practice are essential to comprehensive national programmes of stroke care.

## Background

Stroke is a major cause of mortality[1] and morbidity[2], and might be argued to be a chronic disease with acute events[3]. Population-based data suggest that acute cerebrovascular events are at least as common as coronary events[4]. The lifetime risk of first-ever stroke from age 55 years in the Framingham cohort was as high as 1 in 5[5]. There is considerable opportunity for primary prevention of stroke: treatments for hypertension[6,7] and non-valvular atrial fibrillation[8,9] are effective in reducing the risk of stroke, but may not be fully implemented[10]. Observational studies have shown that lifestyle factors such as diet, smoking, exercise, and alcohol intake can predict the risk of stroke [11-13], thereby supporting the adoption of lifestyle risk factor modification. The actuarial risk of recurrent stroke after a first stroke is about 30% over five years[14], and there is strong evidence of the benefits of anti-platelet therapy[15], blood pressure lowering[16] and lipid lowering[17] in secondary prevention. Despite improvements in the use of secondary prevention medication, there is further scope for achieving more from these medications[18].

General practitioners (GPs) are well placed to implement secondary prevention programmes for stroke. For instance, in an Irish study of 195 discharged stroke patients, the majority (87%) had seen their GP since hospital discharge, whereas just less than half (48%) had been reviewed in hospital outpatient departments[19]. With regard to emergency care, current guidelines recommend all patients with suspected acute stroke are immediately transferred by ambulance to a receiving hospital providing acute stroke services and organised stroke care and that all patients presenting with a recent transient ischaemic attack (TIA) or minor stroke are immediately referred for appropriate urgent specialist assessment and investigation[20]. GPs can play a key role in initiating and directing this rapid response. Finally, GPs can support community-based patient education and primary prevention of stroke since most stroke patients will be community dwellers (for instance, 90% of Irish stroke patients were community-dwelling before being admitted to hospital with a stroke[21]).

In order for GPs to coordinate optimal stroke prevention and care, a structured approach to the detection and management of chronic disease and risk factors is needed[22,23]. Structures to facilitate this include disease registers; clinics for implementing and monitoring the effectiveness of therapy; the use of clinical guidelines or practice protocols to support clinical decisions; and clinical audit to evaluate the effectiveness of treatment provided and stimulate improvement. Information on the capacity of general practice to deliver optimal stroke care is needed. As part of a national evaluation of stroke services in Ireland[24] which assessed community, hospital and nursing home services for stroke, a survey of general practices was undertaken to determine the structures currently in place likely to support stroke prevention and care.

## Methods

### Study design, sampling and participants

We conducted a postal survey among a random sample of GPs taken from a total population of 2,300 GPs in the Republic of Ireland profiled in the Irish Medical Directory 2006–2007 edition. Sampling was conducted using the random selection function in Microsoft Excel. A sample size of 242 GPs provides a 90% probability that prevalence will be within 5% of the true value. Based on an expected 50% response rate, 484 participants were selected.

**Figure 1:**
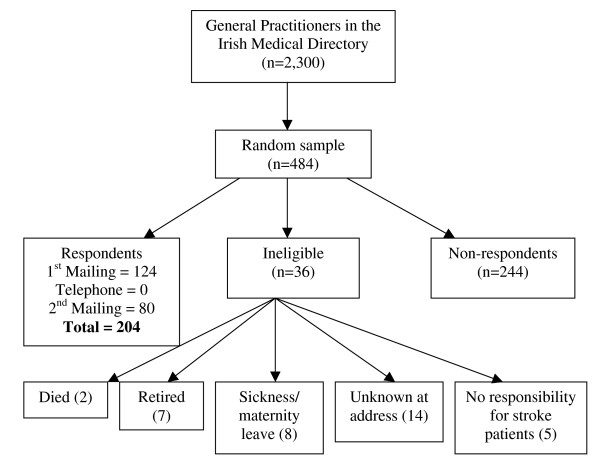


Selected GPs were sent an invitation letter with an explanation of the value of the survey from the study sponsor – the Irish Heart Foundation, a letter of introduction from the research team, the survey instrument, and a stamped addressed envelope. A telephone reminder followed after two weeks if GPs had not returned the questionnaire. GPs were reminded to complete and return the questionnaire, or were given the option to complete the questionnaire by telephone at a time convenient to them. A final reminder questionnaire was sent to non-responders two weeks following the telephone approach (Figure 1). Research ethics approval was granted by the Royal College of Surgeons in Ireland Research Ethics Committee (REC2006:186)

### Questionnaire development

The survey instrument was developed by the research team based on a recent UK general practice survey (Thomas, Chappel, Thomson, & Rogers, personal correspondence, [see Additional file 1]). The content validity and conceptual clarity of the questionnaire was established for the Irish setting through panel discussion and piloting with ten GPs practicing in five general practices in Dublin. The questionnaire included questions on practice organisation, primary prevention of stroke, acute management of stroke, secondary prevention of stroke, long-term care and rehabilitation.

### Analysis

Data analysis was with SPSS version 15.0 using parametric and non-parametric tests of association. P < 0.05 was taken as significant. Variables that were hypothesised to have an impact on delivery of stroke care were membership of Heartwatch (a programme of structured secondary prevention in cardiovascular disease run by 30% of general practices in Ireland), availability of a practice nurse, and being a training practice. Variables respectively associated with each of these variables at the 5% level were entered into a logistic regression analysis.

## Results

Of the 448 GPs who were eligible to participate in the survey, 204 did so (46% response rate). There was no difference between responders and non-responders in terms of gender or practice location (i.e. within Dublin or outside Dublin). However, responders were more likely to have qualified after 1980 than non-responders (X^2 ^= 8.36, 1 df, p = 0.004). The median age of responders was 49 years (inter-quartile range 42–55). A quarter (24%) of the responders was single-handed GPs with the median number of doctors per practice being two (range 1–12). The median practice size was 3,000 patients (range 100–25,000) and the mean was 4,513 (95% CI 3,885–5,141) patients. A quarter of the responders (25%) were in training practices. Response rates to individual questions ranged from 53% to 100% (median 96%) with a mean of 94% (95%CI 92.6–95.2).

### Access to community services

The majority of responders reported good or excellent access to practice nurses (75%), community nurses (71%), and physiotherapists (56%). However, this did not apply to access to other allied health professionals including dieticians (32%), social workers (24%), psychologists (15%), speech and language therapists (13%) and occupational therapists (13%).

### Practice organisation

Practices that were members of the Heartwatch scheme, were training practices and who had practice nurses were more likely to engage in activities likely to prevent strokes (Additional file 2). Variables associated with these three variables respectively were entered into logistic regressions. Membership of the Heartwatch scheme was associated with having registers for atrial fibrillation (Odds ratio (OR) 4.17; 95% CI 1.58–10.21), diabetes (OR 2.68; 95% CI 1.17–6.13), and stroke (OR 3.63; 95% CI 1.19–11.01); and having guidelines for hypertension (OR 2.49; 95% CI 1.07–5.76) and secondary prevention of stroke (OR 5.61; 95% CI 1.77–17.74). Being a member of a training practice was associated with having a computerised disease register (OR 4.17; 95% CI 1.59–10.96), using the computer for audit (OR 4.58; 95% CI 1.73–12.08), having registers for hypertension (OR 4.02; 95% CI 1.58–10.21) and atrial fibrillation (OR 4.17; 95% CI 1.44–12.02) and auditing diabetes care (OR 2.89; 95% CI 1.13–7.41). Being part of a larger practice (>4500 patients) was associated with having registers for hypertension (OR 1.69; 95% CI 1.07–2.68) and diabetes (OR 1.59; 95% CI 1.03–2.46), and carrying out a diabetes audit (OR 6.26; 95% CI 1.19–32.88). Having a practice nurse was associated with having a diabetes register (OR 3.29; 95% CI 1.26–8.58).

### Primary prevention of stroke

Additional file 2 shows aspects of practice structure in relationship to primary prevention of stroke through the management of hypertension, atrial fibrillation and diabetes. Diabetes care was the most organised. The majority of GPs (89%) reported barriers to the implementation of primary prevention for stroke. These included lack of time (89%), inadequate staffing (86%), lack of funding (86%), lack of screening protocols (67%) and lack of risk factor protocols (58%). The main solutions suggested by GPs to overcome these barriers were the employment of practice nurses and the running of dedicated clinics.

Knowledge amongst GPs about primary prevention of stroke was good with over 90% being aware that reduction of blood pressure, reduction of cholesterol, use of aspirin with transient ischaemic attacks (TIA) and anticoagulation in patients with AF, as well as carotid endarterectomy in patients with carotid artery stenosis >70% were effective in the prevention of stroke. However, almost two thirds (65%) of GPs incorrectly believed that anticoagulation in patients with a history of TIA was effective in the prevention of stroke.

### Secondary prevention of stroke

The organisation of services for the secondary prevention of stroke was typically poor (Additional file 2). The main barriers listed for secondary prevention were very similar to those recorded for primary prevention, namely lack of time (60%), inadequate staffing (57%), and issues related to funding (33%). Other barriers listed included lack of protocols/guidelines (17%) and lack of space (10%). 87% of GPs believed that the availability of existing rehabilitation services was inadequate for their stroke patient population.

### Management of stroke

Home management of acute stroke patients was used at some stage by 50% of responders, accounting for 7.3% (95% CI 5.5–9.1) of all stroke patients. However, there was considerable variation among those GPs using home management. The median proportion of patients looked after at home was 10% but this ranged from one to 80% (inter-quartile range 5–20%). Factors influencing the decision for home management included the severity of stroke, the amount of time elapsed since the stroke, the age of the patient, family support, a previous history of stroke and co-morbid disease. Younger GPs (X^2 ^= 12.1, 3 df, p = 0.007), those from larger practices (X^2 ^= 6.0, 1 df, p = 0.014) and those with a lead person for stroke rehabilitation (X^2 ^= 6.9, 1 df, p = 0.008) were less likely to manage patients at home. Almost all GPs (94%) managing acute stroke patients at home used aspirin, but 60% stated they aimed for acute blood pressure reduction after an acute stroke.

Over 85% of GPs reported that there was no routine liaison from hospitals during admission or leading up to discharge. Similarly, 79% reported no liaison following discharge from hospital. However, 98% reported receiving information on discharge medication. Little information was shared with GPs on rehabilitation or community services that had been organised.

## Discussion

This study reveals that there is currently little organisation for the prevention and management of stroke within primary care in Ireland. This is in spite of the clear evidence that up to 60% of stroke mortality is related to a few preventable vascular risk factors[25] and that structured systems of care are delivering improvements in both these risk factors and in the incidence of stroke[26]. Primary care has an enormous potential to deliver primary prevention of stroke but is failing to rise to this challenge in Ireland. However, there are encouraging signs that practices with a higher level of organisation, such as training practices, practices involved in Heartwatch and practices with good access to practice nurses, were more likely to engage in activities that adequately manage stroke patients.

There is not much evidence from this study of the systematic implementation of healthy lifestyle advice and management of specific risk factors needed to reduce the risk of an initial stroke and the risk of a subsequent stroke. Given the high probability of repeat stroke within five years, this lack of secondary prevention and follow-up in general practice is a cause for concern. The study also highlights the need for improvements in the initial management of acute stroke by some GPs.

### Comparison with previous literature

The acute management of stroke by GPs in Ireland compares favorably with that in the UK where just over half of GPs said they would refer someone with a suspected stroke immediately[27]. However, in the UK almost all general practices have a stroke/TIA register and engage in annual audit that has revealed a steady improvement in most preventive measures[28]. The implementation of structures to support the prevention of cardiovascular disease in Ireland through the Heartwatch scheme has shown similar improvements in preventive measures[29]. A previous study has found the quality of stroke care to be significantly related to aspects of practice organisation including delegation to support staff and compliance with hypertension guidelines[23]. These aspects of practice organisation are absent in many general practices in this survey, leaving room for concern at the quality of care received by those surviving stroke.

### Strengths and weaknesses

The response rate in this study was poor but similar to the response rate to many surveys of GPs in Ireland. The smaller sample size provided power of 83%. While the figures in relation to the response rate and power are less than optimal, the study does provide the first national profile from over 200 GPs of stroke management in general practice in Ireland. More recently qualified GPs were over-represented in responders compared to non-responders. As these GPs may have more up-to-date knowledge in relation to chronic disease management, we caution that the results of this survey may reflect a more positive picture of stroke awareness and care in general practice than exists in reality.

### Implications for research and development

Stroke is often the focus of GP consultations, calculated in one study as over 2 per 1000 encounters[30]. However, it is not yet clear how these consultations could be more effectively utilised in the prevention and management of stroke. Equally, the interface between primary and secondary care poses many challenges in stroke care, but studies of hospital-based stroke coordinators working with primary care physicians have not yet shown evidence of efficacy[31]. The basic requirements of chronic disease management are well established and include the three 'R's: register, recall, and review. However, many challenges beyond these basic requirements remain[32]. For instance, this study found that teamwork among health professionals providing primary care was suboptimal, with access to many stroke team members poor or absent, and it is recommended that any structured scheme of stroke care incorporates an interdisciplinary team-based approach. There is a clear need to establish a system of structured stroke care in general practice that will facilitate the implementation of both primary and secondary prevention of stroke. A model for such a scheme already exists in Ireland in the Heartwatch programme[29] and the Quality Outcomes Framework in the United Kingdom provides an alternative approach[28].

## Conclusion

In summary, general practice is well placed to provide primary and secondary prevention of stroke and long-term management of stroke patients. It is not fulfilling this potential in the Irish health system at present. The involvement of primary care in providing a structured approach to stroke management would appear to be essential to any comprehensive national programme of stroke care. It is proposed that an adequately funded and resourced system of structured stroke care is needed in order to address the deficiencies identified in this study.

## Competing interests

The authors declare that they have no competing interests.

## Authors' contributions

All authors participated in the design of the study. FH, AH, BOS and DW participated in the coordination and statistical analysis and drafted the manuscript. HM and DON helped to draft the manuscript. All authors read and approved the final manuscript.

## Pre-publication history

The pre-publication history for this paper can be accessed here:



## Supplementary Material

Additional file 1 **Questionnaire distributed to General Practitioner.** Click here for file

Additional file 2 **Table S1.** Click here for file
