# Supplementary material for: Is primary care a neglected piece of the jigsaw in ensuring optimal stroke care? Results of a national study
Source: BMC Fam Pract. 2009 Apr 29;10:27. doi: 10.1186/1471-2296-10-27 (PMC2680827; doi:10.1186/1471-2296-10-27)
Supplement: Additional file 1 — Questionnaire distributed to General Practitioner. [file 1471-2296-10-27-S1.doc]

#
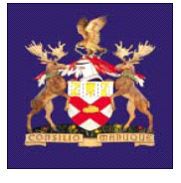

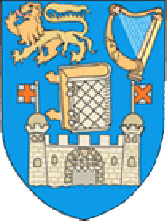


Royal College of Surgeons in Ireland

Trinity College Dublin

#

# Irish Heart Foundation

# National Audit of Stroke Services

# In association with the

# Department of Health and Children

# Management of Stroke by General Practitioners:

# Current Provision and Needs

# Thank you for taking time out to assist us by completing this questionnaire

Demographic information

Male Female

Please state your age

Year of qualification in Medicine MB

**Section 1: Practice details**

| Firstly, we would like to ask a few questions about you and your practice |
| --- |

- 1. How many doctors, including yourself, currently work in your practice?

(a) Full time (b) Part time

1.2 Is your practice a training practice? *(please circle)*  Yes No

1.3 Please rate your practice’s access either on site or via referral to the following health care professionals.

*1 2 3 4 5*

*No access      Very limited         Quite limited                 Good                Excellent*

Practice nurse 1 2 3 4 5

Public health nurse 1 2 3 4 5

Occupational therapist 1 2 3 4 5

Speech and language therapist 1 2 3 4 5

Dietician 1 2 3 4 5

Psychologist 1 2 3 4 5

Counsellor 1 2 3 4 5

Social worker 1 2 3 4 5

Vascular surgeon (in your local acute hospital) 1 2 3 4 5

Community psychiatric nurse 1 2 3 4 5

Other *(please specify)* 1 2 3 4 5

_____________________________________

1.4 Practice category *(please tick the appropriate option below)*

1. Inner city
2. Urban
3. Rural

1.5 What is the approximate distance from your practice to the nearest acute

hospital in miles?

1.6a How many patients are currently in your practice?

1.6b What number of GMS patients do you currently have in your practice?

1.6c What percentage of your practice population are GMS patients?

1.6d How many patients with stroke do you currently provide care for?

1.7a Is your practice computerised? *(please circle)* Yes No

*(If no, please proceed to Section 2: Stroke Population)*

1.7b If computerised**,** for what purpose is the computer generally used?

*(please circle)*

Secretarial/Administration Yes No

Consultations Yes No

`

Prescribing Yes No

Maintain disease register Yes No

Audit Yes No

Other *(please state)* ________________ `

1.8 Are you part of the ICGP Heartwatch scheme? Yes No

**Section 2: Primary prevention of stroke**

| In this section, we would like to ask some questions about your practice’s approach to the prevention of strokes |
| --- |

**2.1 Hypertension**

2.1a Does your practice have a person with an identified lead Yes No

role in hypertension?

2.1b Do you screen routinely for hypertension? Yes No

2.1c Does your practice have a register of patients Yes No

with hypertension?

2.1d Does your practice have guidelines for any of the following?

*(please circle one response option on each line)*

Screening for hypertension Yes No

Diagnosis of hypertension Yes No

Management of hypertension Yes No

Referral for hypertension Yes No

A comprehensive guideline/protocol covering Yes No

all of the above

2.1e Does your practice run a regular hypertension clinic? Yes No

2.1f Has your practice conducted an audit of patients with Yes No

hypertension within the last two years?

**2.2 Atrial fibrillation**

2.2a Does your practice have a person with an identified lead Yes No

role in atrial fibrillation?

2.2b Do you screen routinely for atrial fibrillation? Yes No

2.2c Does your practice have a register of patients Yes No

with atrial fibrillation?

2.2d Does your practice have guidelines for any of the following?

*(please circle one response option on each line)*

Screening for atrial fibrillation Yes No

Diagnosis of atrial fibrillation Yes No

Management of atrial fibrillation Yes No

Referral for atrial fibrillation Yes No

Initiation of warfarin anticoagulation Yes No

Monitoring of warfarin anticoagulation Yes No

Referral for warfarin anticoagulation Yes No

A comprehensive guideline/protocol covering Yes No

all of the above

2.2e Does your practice run an atrial fibrillation clinic? Yes No

2.2f Does your practice run a warfarin clinic? Yes No

2.2g Has your practice conducted an audit of patients with atrial Yes No

fibrillation within the last two years?

**2.3 Diabetes**

2.3a Does your practice have a person with an identified lead Yes No

role in diabetes?

2.3b Do you screen routinely for diabetes? Yes No

2.3c Does your practice have a register of patients Yes No

with diabetes?

2.3d Does your practice have guidelines

for any of the following? *(please circle one response option on each line)*

Screening for diabetes Yes No

Diagnosis of diabetes Yes No

Management of diabetes Yes No

Referral for diabetes Yes No

A comprehensive guideline/protocol covering Yes No

all of the above

2.3e Does your practice run a diabetes clinic? Yes No

2.3f Has your practice conducted an audit of patients with diabetes Yes No

within the last two years?

**2.4 Lifestyle**

2.4a Does your practice have guidelines/protocols for any of the following?:

*(please circle one response option on each line)*

Smoking cessation Yes No

Exercise Yes No

Diet Yes No

Alcohol Yes No

2.4b Does your practice run dedicated clinics for any of the following?:

*(please circle one response option on each line)*

General healthy lifestyle Yes No

Elderly (general) Yes No

Smoking cessation Yes No

Exercise Yes No

Diet / weight reduction Yes No

Alcohol Yes No

Other clinics relevant to stroke Yes No

*(If yes, please specify)*

**2.5 Preventative Measures**

2.5a In your view, are there barriers to implementing primary Yes No

prevention strategies for stroke in your practice?

If yes, can you

1. indicate what these barriers are by ticking the relevant boxes below and indicating additional barriers in the “other” section.
2. Indicate solutions to these barriers

| Barriers | Solutions |
| --- | --- |
| Staffing  Time  Funding  Lack of formal  protocols for screening  Lack of formal protocols  for follow up of risk factors  Other (please indicate any other barriers)  ____________________________________________ | Staffing solutions  Time Solutions  Funding solutions  Protocol Screening solutions  Protocol risk factor solutions  Solutions to other barriers |

2.5b Which of the following would you consider to be effective in the prevention of stroke?

Anticoagulation in patients with history of transient ischemic

attacks (TIA) Yes No

Reduction of blood pressure Yes No

Use of vitamin E Yes No

Reduction of cholesterol Yes No

Use of aspirin in patients with transient ischaemic attacks (TIA) Yes No

Anticoagulation in patients with atrial fibrillation (AF) Yes No

Carotid endarterectomy in patients with carotid artery stenosis (> 70%) Yes        No

**3. Management of stroke**

| In this section we would like to ask some questions about your practice’s management of stroke patients |
| --- |

3.1a Do you have a stroke register? *(please circle)* Yes No

*(If your answer is ‘No’ please proceed to Question 3.2*)

3.1b If yes, is your existing stroke register computerised? Yes No

3.2 Does your practice have a person with an identified lead Yes No

role in stroke?

3.3 Does your practice run a stroke clinic? Yes No

3.4 Has your practice conducted an audit of patients with stroke Yes No

within the last two years?

3.5 Does your practice have guidelines for any of the following? *(please circle one response option on each line)*

Diagnosis of acute management of stroke Yes No

Management of acute stroke Yes No

Referral for acute stroke Yes No

3.6 Which of the following would you consider to be effective in the acute management of patients with ischaemic stroke?

Specialised stroke rehabilitation Yes No

Aspirin Yes No

Immediate systemic anticoagulation Yes No

Nifedipine Yes No

Piracetam Yes No

Thrombolysis Yes No

3.7 In the acute management of stroke, can you estimate the percentage of **your** patients that are initially managed using the following methods:

Home management % Immediate transfer to hospital %

*If* ***any*** *of your patients are managed at home, please proceed with question 3.8. If none of your patients are managed at home, please proceed to question* ***3.12*** *Hospital discharge*

3.8 What factors influence your decision to opt for stroke management at home?

Severity of stroke Yes No

Time since stroke Yes No

Age of patient Yes No

Distance from hospital Yes No

Family support Yes No

Previous history of stroke Yes No

Co morbid disease Yes No

Other (please specify) Yes No

*____________________________________________________*

3.9 Do you currently have direct access to any of the following diagnostic

facilities?

ECG Yes No

MRI Yes No

CT Scan Yes No

Other *(please state)*

*___________________________________________________*

3.10 What therapies do you use in the home management of acute ischaemic stroke? *(please circle)*

Aspirin Yes No

Thrombolysis Yes No

Acute blood pressure reduction Yes No

Corticosteroids Yes No

Other (please specify)

___________________________________________________

3.11 This question contains three components:

Which of the following health care professionals do your patients managed at home typically:

(i) Need

(ii) Have access to

1. Pay for

Please circle an answer for **each** of the three components for **each** profession.

(i) Need (ii) Have access to (iii) Pay for

Practice nurse Yes No Yes No Yes No

Public health nurse Yes No Yes No Yes No

Physiotherapist Yes No Yes No Yes No

Occupational therapist Yes No Yes No Yes No

Speech and language therapist Yes No Yes No Yes No

Dietician Yes No Yes No Yes No

Psychologist Yes No Yes No Yes No

Counsellor Yes No Yes No Yes No

Social Worker Yes No Yes No Yes No

Other Yes No Yes No Yes No

*(please specify)*

**___________________________**

**3.12 Hospital discharge**

3.12a PRIOR to the hospital discharge of your patients with stroke, Yes No

does the hospital medical team routinely liaise with you?

3.12b Are you sent information on patients who Yes No

have had a stroke immediately prior to discharge?

3.12c When a patient is discharged from hospital with a stroke, which of the following types of information are typically provided to you? *(please circle one response option on each line)*

Details of stroke type Yes No

Details of stroke severity Yes No

Functional ability Yes No

Rehabilitation services Yes No

Medications Yes No

Diagnostic test results Yes No

Home help * Yes No

Home care attendant* Yes No

Meals on wheels Yes No

Personal assistant* Yes No

Other *(please specify)* Yes No

_________________________________________________________

**** Definitions for Q3.12 c and Q5.6***

**Home helps**:  may be employed either by the HSE or by voluntary organisations. They assist with normal household tasks and are assigned to people who are unable to carry out such tasks themselves. Availability varies greatly from place to place and there may be a small charge.

**Home care attendant**: They provide assistance and support to people with physical disabilities in their own homes. The time the attendant spends in each person’s home and the tasks carried out vary from family to family.

**Personal assistants**: They enable people to live independently in the community. The Personal Assistant may provide assistance with bathing, dressing, cooking or other personal or household tasks. They may also assist the person in going to and from work, may aid him/her in working or studying or participating in social life. A Personal Assistant can assist a person with a vision impairment with, for example, reading mail, getting from A to B or shopping independently. The person with a disability agrees the range of tasks with the Personal Assistant.

3.12d In the period FOLLOWING hospital discharge of a stroke Yes No

patient, does the medical team routinely liaise with you?

3.12e Do you receive notification from the hospital indicating the Yes No

point at which a patient is FINALLY DISCHARGED from the

hospital, i.e. has no further medical out patient appointments?

**Section 4: Secondary prevention of stroke**

| In this section we would like to ask you about secondary prevention of stroke in your practice |
| --- |

4.1 Do you have a person with an identified lead role in Yes No

secondary prevention of stroke?

4.2 Does your practice have written guidelines/protocols for any of the following *(please circle one response option on each line)*

Antiplatelet therapy (e.g. aspirin, dipyridamole) for ischaemic Yes No

heart disease

Antiplatelet therapy (e.g. aspirin, dipyridamole) for stroke Yes No

disease

Assessment of vascular risk Yes No

Follow up of stroke patients at high risk of a further stroke Yes No

Secondary prevention of stroke in general Yes No

4.3 Does your practice run a secondary Yes No

prevention clinic?

4.4 Does your practice run a TIA clinic? Yes No

4.5 Has your practice conducted any of the following stroke-related activities? *(please circle*)

(i) An audit within the last 2 years of stroke patients Yes No

(ii) An audit within the last 2 years of TIA patients Yes No

4.6 Please rate how often you provide information on the following lifestyle modifications to stroke patients using the scale: *(please circle one response option on each line)*

*1 Always 2 Frequently 3 Sometimes 4 Occasionally 5 Never*

Weight management 1 2 3 4 5

Diet/healthy nutrition 1 2 3 4 5

Reducing salt intake 1 2 3 4 5

Participation in regular physical activity 1 2 3 4 5

Avoidance of excessive alcohol consumption 1 2 3 4 5

Smoking cessation 1 2 3 4 5

Adherence to medication 1 2 3 4 5

4.7 In your view, are there barriers to implementing secondary Yes No

prevention strategies for stroke in your practice?

If yes, can you

1. indicate what these barriers are below
2. Indicate solutions to these barriers

| Barriers | Solutions |
| --- | --- |
|  |  |

**Section 5: Stroke rehabilitation and long term care**

| In this section, we would like to ask some questions about your practice’s approach to stroke rehabilitation and long term care |
| --- |

5.1 Does your practice have a person with an identified Yes No

lead role in stroke rehabilitation and/or long term care for

stroke patients?

5.2 Does your practice have guidelines or protocols for Yes No

stroke patient rehabilitation?

5.3 Does your practice have guidelines on information to be given Yes No

to patients and/or carers?

5.4 Do you receive communication regarding rehabilitation Yes No

services that have been organized for the stroke patient

following discharge from hospital?

5.5 When a patient is receiving ongoing rehabilitation following discharge from hospital, where is the rehabilitation generally carried out?

(a) Hospital Yes No

(b) Community Yes No

(c) Other ________________

- 1. This question consists of three components:

Which of the following health care professionals do your patients, post-discharge from hospital typically:

(i) Need

(ii) Have access to

(iii) Pay for

*(please answer yes or no for EACH profession)*

**(i) Need (ii) Have access to (iii) Pay for**

Personal assistant service Yes No Yes No Yes No

Home care attendant Yes No Yes No Yes No

Home help Yes No Yes No Yes No

Physiotherapist Yes No Yes No Yes No

**(i) Need (ii) Have access to (iii) Pay for**

Occupational therapist Yes No Yes No Yes No

Speech & language therapist Yes No Yes No Yes No

Psychologist Yes No Yes No Yes No

Counsellor Yes No Yes No Yes No

Social Worker Yes No Yes No Yes No

Other Yes No Yes No Yes No

*(please specify)*

_______________________________

5.7a Can you please estimate the proportion of your stroke patients who are clinically depressed? _____________%

5.7b Of those depressed, what proportion do you estimate were/are depressed:

- before the stroke ____%

- as a consequence of the stroke ____%

5.7c What proportion are currently treated specifically for depression:

- antidepressant or other medication _____%

- counseling/psychological therapy ­ funded by practice or HSE ____%

- counseling/psychological therapy ­ funded by patient ____%

5.7d What proportion of your stroke patients, who need treatment, are not currently treated for depression?____%

5.7e What are the main barriers/solutions to treatment?

| Barriers | Solutions |
| --- | --- |
|  |  |

5.8a Is the availability of existing rehabilitation services, adequate for your stroke patient population? Yes No

5.87b If no, can you indicate

(i) what are the priority areas that need to be addressed?

(ii) possible solutions to these priority areas?

| Barriers | Solutions |
| --- | --- |
|  |  |

5.9a Do you have any stroke patients who reside in a nursing home? Yes No

(If you answered No, please proceed to section 6)

5.9b Please indicate the percentage of your stroke patients that reside in nursing homes. ___

5.9c For what percentage of these patients do you continue to provide medical care?____

5.9d For patients with stroke residing in nursing homes, how is the relationship with community rehabilitation services managed?

5.9e Do you encounter any challenges that are specific to nursing home residence i.e., that are not experienced when patients reside elsewhere? If yes, please explain.

**Section 6: Information and education**

In this section, we would like to ask you about your current information use and needs

**6.1 Where do you currently access educational information on stroke**?

|  | Please tick appropriate box | Please rate usefulness (1-4)  1 Very useful  2 Quite useful  3 Slightly useful  4 Not useful at all |
| --- | --- | --- |
| CME Network |  |  |
| ICGP |  |  |
| Irish Heart Foundation |  |  |
| Heartwatch |  |  |
| Medical journals |  |  |
| Medical newspapers |  |  |
| Internet |  |  |
| Others *(please state)* |  |  |

**6.2 Can you please indicate any stroke-related information needs you have that are currently not being addressed?**

**THANK YOU FOR COMPLETING THIS QUESTIONNAIRE**
