# Supplementary material for: Is primary care a neglected piece of the jigsaw in ensuring optimal stroke care? Results of a national study
Source: BMC Fam Pract. 2009 Apr 29;10:27. doi: 10.1186/1471-2296-10-27 (PMC2680827; doi:10.1186/1471-2296-10-27)
Supplement: Additional file 2 — Table S1. [file 1471-2296-10-27-S2.doc]

**Table 1**

| **Practice criterion** | **GPs with good access to practice nurse** | **GPs with poor access to practice nurse** | **Statistic** | **Member of Heartwatch** | **Not member of Heartwatch** | **Statistic** | **Training practice** | **Non-training practice** | **Statistic** |
| --- | --- | --- | --- | --- | --- | --- | --- | --- | --- |
| **Maintains computerised disease register** | 64/128 (50%) | 5/31 (16%) | Χ^2^ = 11.66, 1df, p<0.001 | 65/66 (98%) | 97/121 (80%) | Χ^2^ = 12.37, 1df, p<0.001 | 28/42 (67%) | 32/99 (32%) | Χ^2^ = 14.23, 1df, p<0.001 |
| **Uses computer for clinical audit** | 50/124 (40%) | 3/30 (10%) | Χ^2^ = 9.84, 1df, p=0.002 | 30/61 (49%) | 21/86 (24%) | Χ^2^ = 9.66, 1df, p=0.002 | 25/47 (60%) | 21/99 (21%) | Χ^2^ = 18.28, 1df, p<0.001 |
| **Has register of patients with hypertension** | 51/147 (35%) | 8/50 (16%) | Χ^2^ = 6.21, 1df, p=0.013 | 30/66 (45%) | 26/121 (21%) | Χ^2^ = 11.69, 1df, p=0.001 | 24/45 (53%) | 31/134 (23%) | Χ^2^ = 14.43, 1df, p<0.001 |
| **Has register of patients with atrial fibrillation** | 31/147 (21%) | 3/49 (6%) | Χ^2^ = 5.74, 1df, p=0.017 | 20/66 (30%) | 12/120 (10%) | Χ^2^ = 12.32, 1df, p<0.001 | 16/45 (36%) | 16/133 (12%) | Χ^2^ = 12.62, 1df, p<0.001 |
| **Has register of patients with diabetes** | 88/144 (61%) | 12/51 (24%) | Χ^2^ = 21.29, 1df, p<0.001 | 48/66 (73%) | 49/119 (41%) | Χ^2^ = 16.94, 1df, p<0.001 | 34/44 (77%) | 59/133 (44%) | Χ^2^ = 14.36, 1df, p<0.001 |
| **Has stroke register** | 24/144 (17%) | 3/50 (6%) | Χ^2^ = 3.52, 1df, p=0.06 | 15/65 (23%) | 10/119 (8%) | Χ^2^ = 7.71, 1df, p=0.005 | 9/44 (20%) | 15/132 (11%) | Χ^2^ = 2.32, 1df, p=0.13 |
| **Hypertension guidelines** | 34/139 (24%) | 10/47 (21%) | Χ^2^ = 0.20, 1df, p=0.66 | 41/64 (64%) | 65/117 (56%) | Χ^2^ = 1.23, 1df, p=0.27 | 27/43 (63%) | 76/130 (58%) | Χ^2^ = 0.25, 1df, p=0.62 |
| **Has guidelines on initiation of warfarin in atrial fibrillation** | 48/145 (33%) | 6/49 (12%) | Χ^2^ = 7.93, 1df, p=0.005 | 22/66 (33%) | 28/119 (23%) | Χ^2^ = 2.07, 1df, p=0.15 | 18/45 (40%) | 31/133 (23%) | Χ^2^ = 4.7, 1df, p=0.03 |
| **Acute stroke guidelines** | 24/139 (17%) | 13/47 (28%) | Χ^2^ = 2.38, 1df, p=0.12 | 12/63 (19%) | 24/115 (21%) | Χ^2^ = .084, 1df, p=0.77 | 6/44 (14%) | 27/125 (22%) | Χ^2^ = 1.31, 1df, p=0.25 |
| **Secondary prevention of stroke guidelines** | 24/143 (17%) | 12/46 (26%) | Χ^2^ = 1.95, 1df, p=0.16 | 16/64 (25%) | 19/115 (16%) | Χ^2^ = 1.88, 1df, p=0.17 | 8/43 (19%) | 26/128 (21%) | Χ^2^ = 0.06, 1df, p=0.81 |
| **Runs a warfarin clinic** | 66/147 (45%) | 8/48 (17%) | Χ^2^ = 12.25, 1df, p<0.001 | 31/66 (47%) | 36/118 (30%) | Χ^2^ = 4.95, 1df, p=0.026 | 25/45 (56%) | 44/131 (34%) | Χ^2^ = 6.78, 1df, p=0.009 |
| **Runs a diabetes clinic** | 44/145 (30%) | 5/51 (10%) | Χ^2^ = 8.49, 1df, p=0.004 | 25/66 (38%) | 22/120 (18%) | Χ^2^ = 8.61, 1df, p=0.003 | 20/44 (45%) | 26/134 (19%) | Χ^2^ = 11.73, 1df, p=0.001 |
| **Screens routinely for atrial fibrillation** | 81/147 (55%) | 36/49 (73%) | Χ^2^ = 5.15, 1df, p=0.023 | 29/66 (44%) | 79/120 (66%) | Χ^2^ = 8.38, 1df, p=0.004 | 21/45 (47%) | 88/133 (66%) | Χ^2^ = 5.38, 1df, p=0.02 |
| **Audits hypertension care** | 11/146 (8%) | 2/50 (4%) | Χ^2^ = 0.75, 1df, p=0.39 | 11/66 (17%) | 2/120 (2%) | Χ^2^ = 14.74, 1df, p<0.001 | 7/44 (16%) | 5/134 (4%) | Χ^2^ = 7.81, 1df, p=0.005 |
| **Audits atrial fibrillation care** | 6/141 (4%) | 3/49 (6%) | Χ^2^ = 0.35, 1df, p=0.55 | 6/66 (9%) | 4/120 (3%) | Χ^2^ = 2.77, 1df, p=0.10 | 3/45 (7%) | 6/133 (4%) | Χ^2^ = 0.32, 1df, p=0.57 |
| **Audits diabetes care** | 34/145 (23%) | 6/51 (12%) | Χ^2^ = 3.17, 1df, p=0.075 | 20/66 (30%) | 20/120 (17%) | Χ^2^ = 4.69, 1df, p=0.003 | 18/44 (41%) | 19/134 (14%) | Χ^2^ = 14.37, 1df, p<0.001 |
| **Audits stroke care** | 3/142 (2%) | 3/49 (6%) | Χ^2^ = 1.92, 1df, p=0.16 | 3/64 (5%) | 3/117 (3%) | Χ^2^ = 0.58, 1df, p=0.45 | 0/44 (0%) | 5/130 (4%) | Χ^2^ = 1.74, 1df, p=0.19 |
